# Tumor-associated stromal cells as key contributors to the tumor microenvironment

**DOI:** 10.1186/s13058-016-0740-2

**Published:** 2016-08-11

**Authors:** Karen M. Bussard, Lysette Mutkus, Kristina Stumpf, Candelaria Gomez-Manzano, Frank C. Marini

**Affiliations:** 1 Department of Cancer Biology, Wake Forest Comprehensive Cancer Center, Winston-Salem, NC 27157 USA; 2 Department of Cancer Biology, Sidney Kimmel Cancer Center, Thomas Jefferson University, Philadelphia, PA 19107 USA; 3 Department of Regenerative Medicine, Wake Forest University, Winston-Salem, NC 27157 USA; 4 Department of Neuro-Oncology, The University of Texas MD Anderson Cancer Center, Houston, TX 77030 USA

**Keywords:** Tumor-associated fibroblast, Cancer-associated fibroblast, Mesenchymal stem cell, Myofibroblast, Stroma, Tumor microenvironment, Tumor-associated stroma, Alpha-smooth muscle actin, microRNA, Exosome, IL-6, MCP-1

## Abstract

The tumor microenvironment is a heterogeneous population of cells consisting of the tumor bulk plus supporting cells. It is becoming increasingly evident that these supporting cells are recruited by cancer cells from nearby endogenous host stroma and promote events such as tumor angiogenesis, proliferation, invasion, and metastasis, as well as mediate mechanisms of therapeutic resistance. In addition, recruited stromal cells range in type and include vascular endothelial cells, pericytes, adipocytes, fibroblasts, and bone-marrow mesenchymal stromal cells. During normal wound healing and inflammatory processes, local stromal cells change their phenotype to become that of reactive stroma. Under certain conditions, however, tumor cells can co-opt these reactive stromal cells and further transition them into tumor-associated stromal cells (TASCs). These TASCs express higher levels of proteins, including alpha-smooth muscle actin, fibroblast activating protein, and matrix metalloproteinases, compared with their normal, non-reactive counterparts. TASCs are also known to secrete many pro-tumorigenic factors, including IL-6, IL-8, stromal-derived factor-1 alpha, vascular endothelial growth factor, tenascin-C, and matrix metalloproteinases, among others, which recruit additional tumor and pro-tumorigenic cells to the developing microenvironment. Here, we review the current literature pertaining to the origins of recruited host stroma, contributions toward tumor progression, tumor-associated stromal cells, and mechanisms of crosstalk between endogenous host stroma and tumor cells.

## Background

The tumor microenvironment is a heterogeneous population of cells composed of tumor cells plus nearby endogenous stromal cells recruited by the tumor [1]. It is becoming well established that, during tumor progression, the tumor cell “seed” co-evolves with the surrounding microenvironment “soil” and that there is substantial crosstalk between the various cell types which promote tumor growth and development [2]. These supporting cells, recruited from the local host stroma, promote extracellular matrix remodeling, cellular migration, neoangiogenesis, invasion, drug resistance, and evasion of immunosurveillance through production of various growth factors, chemokines, and cytokines [2]. Though stromal composition is known to vary between tumors [1], little is known about a) the recruitment process by which tumor cells co-opt the host stroma, or b) mechanisms of crosstalk between the host stroma and tumor cells. Here, we review the current literature pertaining to the origins of recruited host stroma, contributions toward tumor progression, tumor-associated fibroblasts, and mechanisms of crosstalk between endogenous host stroma and tumor cells.

### Origins of tumor-recruited stroma

**Fig. 1:**
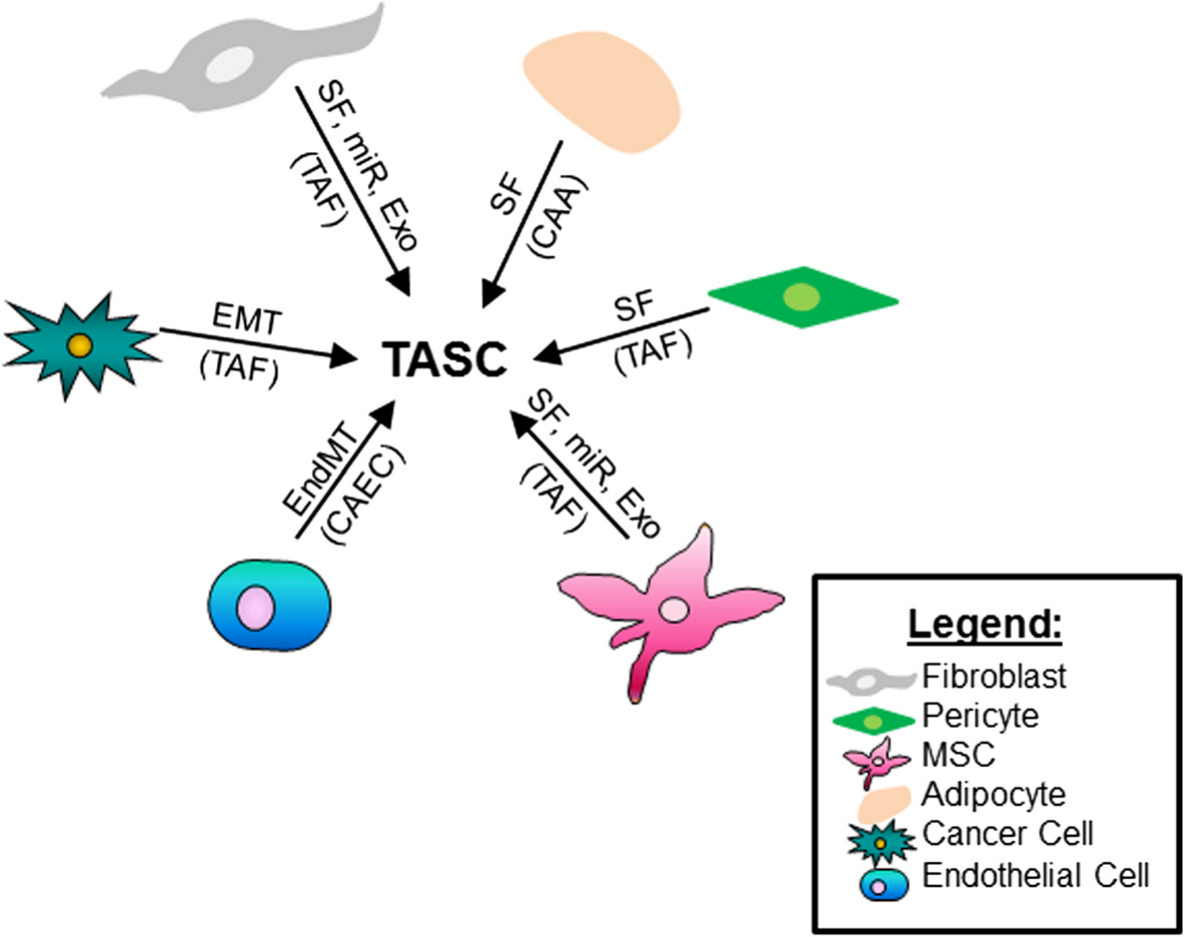
Tumor-associated stromal cells arise from distinct cellular sources. Tumor-associated stromal cells (*TASC*) have been found to arise from at least six distinct cellular origins: fibroblasts, pericytes, bone marrow MSCs, adipocytes, endothelial cells that have undergone an endothelial mesenchymal transition (*EndMT*), or tumor cells that have undergone a epithelial to mesenchymal transition (*EMT*). Transition of these cells occurs via soluble factors (*SF*), microRNAs (*miR*), exosomes (*Exo*), EMT, or EndMT and results in the formation of the TASC subtypes: tumor-associated fibroblasts (*TAF*), cancer-associated adipocytes (*CAA*), or cancer-associated endothelial cells (*CAEC*)

Interactions between the host stroma and tumor cells play a critical role in tumor growth and progression. As described by Dvorak [3], tumor stromal generation exhibits many similarities to normal wound healing, including neoangiogenesis, infiltration of fibroblasts and immune cells, and extensive remodeling of the extracellular matrix. Although these events facilitate the production of the tumor bulk, tumors are strikingly heterogeneous in their overall composition. This is primarily due to the recruitment of nearby non-cancerous host stromal cells, including bone-marrow mesenchymal stromal cells (MSCs), adipocytes, and endothelial cells, that secrete a plethora of mediators and growth factors for the tumor that help facilitate tumor progression [3]. At the present, several sources of host tissue have been identified as targets for stromal cell recruitment by tumors: bone marrow, composed of mesenchymal cells, endothelial cells, immune cells, adipocytes, and fibroblasts; connective tissue, composed of fibroblasts and mesenchymal cells; adipose tissue, composed of adipocytes; and blood vessels, composed of pericytes and endothelial cells [1, 4]. In fact, recent data have indicated that tumor-associated stroma are a prerequisite for tumor cell invasion and metastasis and arise from at least six distinct cellular origins: fibroblasts [5], pericytes [6], bone marrow MSCs [6], adipocytes [4], macrophages [7], and immune cells [8] (Fig. 1).

Within the tumor microenvironment, there is substantial evidence of cellular transdifferentiation, both from stromal cell to stromal cell and from tumor cell to stromal cell. The most frequently cited example is that of fibroblast transdifferentiation into activated myofibroblast during formation of the reactive stroma [9]. Evidence has been provided suggesting that this phenomenon is both a transdifferentiation event [10] and a differentiation event [9], depending on the circumstances. Other examples suggest evidence for pericyte transdifferentiation into endothelial cells or fibroblasts, capable of forming tumor-associated stromal cells (TASCs) [11]. On the other hand, evidence suggests that cancer cells are capable of transdifferentiation into stromal-like cells in order to facilitate tumor progression. Scully et al. [12] found that glioblastoma stem-like cells were capable of transdifferentiation into mural-like endothelial cells in order to promote vascular mimicry. Furthermore, Twist 1 was found to promote endothelial cell transdifferentiation of head and neck cancer cells via the Jagged1/KLF4 axis in order to enhance tumor angiogenesis [13]. Most recently, Cerasuolo et al. [14] discovered that androgen-dependent LNCaP cells cultured long-term in hormone independent conditions permitted the transdifferentiation of prostate cancer cells into a non-malignant neuroendocrine cell phenotype, which were subsequently able to support the growth of additional androgen-dependent prostate cancer cells in the tumor microenvironment.

**Fig. 2:**
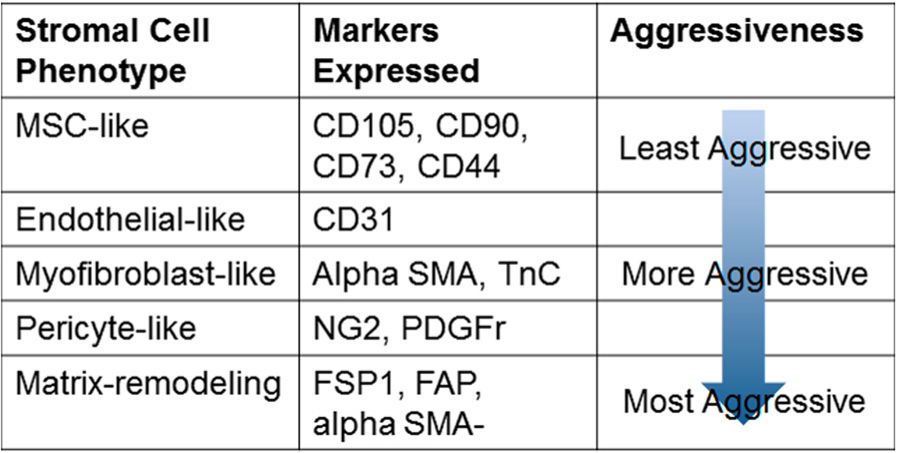
Continuum of tumor-associated stromal cell phenotypes. We propose the existence of at least five tumor-associated fibroblast subtypes as distinguished by specific markers during the course of tumor progression: *MSC-like* is the least aggressive as evidenced by lack of remodeling of the extracellular tumor matrix and expression of MSC markers CD105, CD90, CD73, and CD44; *Endothelial-like* cells, which express CD31; *Myofibroblast-like*, which are more aggressive “activated” stroma and express alpha-smooth muscle actin (alpha SMA) and tenascin C (TnC); *Pericyte-like*, which express NG2 and platelet-derived growth factor receptor (PDGFr); and *Matrix-remodeling*, which are the most aggressive subtype indicated by extensive tumor matrix remodeling, increased expression of FAP and FSP1, and decreased expression of alpha-SMA

We and others have demonstrated that the cellular origin of tumor-associated stroma may shape the phenotypic and biological characteristics of TASCs and, in turn, contribute to the appearance of tumor-associated stroma as a heterogeneous cell population with distinct subtypes that express specific cellular markers [1]. These characteristics are indicated in a hierarchical clustering scheme in Fig. 2. At present, our laboratory has identified at least five tumor-associated stroma subtypes of fibroblastic cells (data not published) ranging from “mesenchymal stem cell-like” (the least aggressive TASC as evidenced by lack of remodeling of the extracellular matrix and expression of MSC markers CD105, CD90, CD73, and CD44) to the most aggressive “matrix remodeling” subtype indicated by extensive tumor matrix remodeling and increased expression of fibroblast activating protein (FAP) and fibroblast specific protein-1 (FSP1), but decreased expression of alpha-smooth muscle actin (alpha-SMA) [1, 15] (data not shown).

### TASC subtypes

#### Nomenclature

Within the tumor microenvironment, several cell types have been the focus of attention, including fibroblasts, myofibroblasts, pericytes, endothelial cells, macrophages, dendritic cells, and other immune cells. Standard nomenclature for the fibroblastic populations vary between tumor-associated fibroblasts (TAFs), cancer-associated fibroblasts (CAFs), carcinoma-associated fibroblasts (also collectively labeled as CAFs), and tumor/cancer-associated stromal cells (TASC/CASC). In the field, however, many of these terms are used interchangeably, which can lead to confusion. In most cases, at least one of several markers is used to characterize the “reactive stroma”, frequently defined as TAF/CAF/TASC/CASC. However, we propose that there is a distinct difference between the acronyms for cancer-associated fibroblast, carcinoma-associated fibroblast, and tumor-associated fibroblast. To illustrate this difference, we provide the definitions of the three words, cancer, carcinoma, and tumor: 1) cancer refers to a disease caused by cells that are not normal and that can spread to one or many parts of the body; 2) carcinoma refers to a malignant tumor of epithelial origin; and 3) tumor refers to an abnormal benign or malignant new growth of tissue that possesses no physiological function and arises from uncontrolled, usually rapid cellular proliferation [16]. From these definitions, we postulate the following: 1) a cancer-associated fibroblast is one that is exposed to disease (cancer) but can be found in any location within the body associated with that disease or its spread (For the remainder of this publication, the term "CAF" refers to "Cancer-Associated Fibroblast."); 2) a carcinoma-associated fibroblast is one that can be found in direct contact with a tumor of epithelial origin, thus excluding hematological malignancies, sarcomas, germ-cell tumors, and all other non-epithelial tumors; and 3) a tumor-associated fibroblast is one that can be found in direct contact with, or immediately adjacent to, a tumor. Furthermore, we propose that TAFs, CAFs, and other tumor-associated cells can all be classified under the heading of “tumor-associated stromal cells” (TASCs).

#### TAFs/CAFs

Fibroblasts regulate the structure and function of healthy tissues via extracellular matrix remodeling and transient tissue repair during wound healing [17]. However, a growing body of evidence demonstrates that fibroblasts are key players in tumorigenesis and constitute the majority of stromal cells within a tumor, especially in breast, prostate, and pancreatic cancers [17]. TAFs/CAFs are activated fibroblasts that share many similarities with normal fibroblasts found during wound healing and inflammation [18]. During tumor progression, TAFs/CAFs show increased rates of proliferation, promote tumor growth via a variety of mechanisms, and mediate therapeutic resistance [18]. In a study by Erez et al. [19], TAFs/CAFs in the tumor stroma promoted sustained inflammation via increase of inflammatory cytokines, neoangiogenesis, and macrophage recruitment, enhancing tumor growth. TAFs/CAFs are also known to enhance angiogenesis via secretion of factors that stimulate pericytes and endothelial cells and have also been implicated in extracellular matrix remodeling [2]. In the past, MSC- and fibroblast-derived TAFs/CAFs have been defined by a specific subset of markers, including alpha-SMA, tenascin C, fibroblast-specific protein-1, fibroblast activing protein, and neural-glial antigen [20]. However, the different sources of TAFs/CAFs, cellular heterogeneity of the tumor microenvironment, similarity of TAFs/CAFs to normal host fibroblasts, as well as inconsistencies in nomenclature make it difficult to distinguish TAFs/CAFs in the tumor stroma from other cell types expressing similar markers. Thus, there is a need for a well-defined list of TASC subtypes, complete with their cellular markers as well as tissue of origin.

#### Cancer-associated adipocytes

In addition to CAFs/TAFs, there is growing evidence to support a TASC subtype derived exclusively from adipose tissue called cancer-associated adipocytes (CAAs) [4] (Fig. 1). Found at the invasive front of tumors, CAAs have been shown to express factors involved in matrix remodeling, invasion and survival of cancer cells, as well as induce epithelial to mesenchymal transition (EMT) [4]. In particular, Wang et al. [21] discovered that CAAs produced increased amounts of insulin-like growth factor binding protein-2 (IGFBP-2) compared with their normal adipocyte counterparts and that this CAA-derived IGFBP-2 resulted in enhanced migration and metastasis of human breast cancer cells both in vitro and in vivo. Furthermore, Dirat et al. [22] showed that mature adipocytes co-cultured with breast cancer cells increase their expression of matrix metalloproteinases (MMP-11) as well as the pro-inflammatory cytokines interleukin (IL)-6 and IL-1β. And Nieman et al. [23] demonstrated that co-culture of human adipocytes with ovarian cancer cells led to increased adipocyte production of IL-8 and fatty acid-binding protein 4, which were found to promote the homing, migration, and invasion of ovarian cancer cells. Co-culture of the adipocytes and ovarian cancer cells was additionally found to stimulate lipolysis in the adipocytes as well as β-oxidation in the ovarian cancer cells, suggesting that the CAAs may additionally be an energy source for the cancer cells. These data as a whole suggest that crosstalk between adipocytes and cancer cells result in the formation of CAAs, which promote the homing and metastasis of cancer cells as well as participate in the development of the tumor microenvironment.

#### Cancer-associated endothelial cells

In addition to CAFs/TAFs and CAAs, an endothelial cell-derived TASC subtype is also known to play an important role in tumor cell growth and invasion. Data have shown that proliferating endothelial cells derived from the bone-marrow undergo an endothelial-to-mesenchymal transition (EndMT) in the presence of tumor growth factor (TGF)-beta, converting the endothelial cells into fibroblast-like cells [23] (Fig. 1). These newly derived cancer-associated endothelial cells (CAECs) exhibit down-regulation of endothelial cell markers CD31 and up-regulation of the TAF/CAF markers FSP1 and alpha-SMA [23]. Interestingly, breast cancer treatment with chemotherapeutic agents has been found to increase CAEC-derived production of tumor necrosis factor (TNF)-alpha, causing an increase in production of CXCL1/2 via nuclear factor (NF)-kappaB by the cancer cells [24]. Increased CXCL1/2 production both attracts myeloid cells and causes them to increase their production of S100A8/9 proteins, which increase breast cancer cell survival and chemoresistance [24]. Other groups have described a type of cancer-activated circulating endothelial cell that was found to promote tumor cell metastasis and protect tumor cells in circulation from targeted therapeutics via chaperoning cancer cells to distant sites [25]. Thus, these results suggest that CAECs are key players in cancer cell evasion of immunosurveillance and enhanced chemoresistance.

#### Tumor-associated immune stroma

Immune cells have also been identified as contributing to the tumor-associated microenvironment via dysregulation of immune-mediated responses. Macrophages, dendritic cells, natural killer (NK) cells, myeloid-derived suppressor cells, and regulatory T cells (T_regs_) have all been shown to contribute toward the polarization of a pro-tumorigenic microenvironment due to their functional responses to contextual cues within the tumor niche. Briefly, tumor-associated macrophages (TAMs) are a distinct M2-polarized macrophage population that promote immune-suppression, pro-angiogenesis, and tumor cell migration and invasion [7]. Targeting of TAMs leads to reduced tumor cell invasion, angiogenesis, and metastasis, as well as enhance the antitumor activity of chemotherapeutics [26]. Myeloid-derived suppressor cells have been shown to differentiate into TAMs and dendritic cells during tumor progression and contribute to tumorigenesis through enhancement of tumor immune evasion, matrix remodeling, and tumor cell EMT [27]. Dendritic cell activity is frequently dysregulated in cancer, leading to reduction in mature dendritic cell numbers, abnormal maturation (and increased numbers of immature dendritic cells with tolerogenic and immunosuppressive capabilities), and suppressed differentiation [28]. Two distinct NK subpopulations, called tumor-infiltrating natural killer cells (TINKs) and tumor-associated natural killer cells (TANKs), have been described in tumor tissues [29]. These NK subpopulations exhibit altered cytokine expression, including increased levels of pro-angiogenic factors such as vascular endothelial growth factor (VEGF) and stromal-derived factor-1 (SDF-1), leading to sustained angiogenesis and tumor progression [30]. Finally, T_regs_ have been shown to play a causal role in tumor progression via infiltration of tumor tissue and reduction of the antitumor immune response [31]. Furthermore, Facciabene et al. [32] recently reported that T_regs_ produced VEGF-A, leading to sustained angiogenesis in a mouse model of ovarian cancer. Taken together, this evidence suggests that contextual responses of immune cells within the tumor stroma helps to drive tumor progression.

### TASCs and soluble factor signaling within the tumor microenvironment

As previously described, TASCs have been found to secrete a variety of soluble factors that enhance formation of the tumor microenvironment. These factors include pro-inflammatory cytokines (IL-6, IL-8, IL-1β, to name a few), matrix metalloproteinases, and growth factors, among others. In the next few paragraphs, we focus on a small sampling of these TASC-derived factors as examples (the pro-inflammatory cytokines and chemokines IL-6, CXCL1, IL-1β, and TNF-alpha) and discuss their contribution towards signaling in the tumor microenvironment and stimulation of additional TASC-derived factors in detail.

#### IL-6

TASCs promote tumor growth in the microenvironment via secretion of cytokines and chemokines. IL-6, specifically, is a pro-inflammatory cytokine known to alter stromal cell function, migration, and EMT in the tumor microenvironment [33]. Importantly, Osuala et al. [34] determined that TAFs, in particular, produce large amounts of IL-6, which promotes the growth and proliferation of MCF10.DCIS ductal carcinoma in situ (DCIS) breast cancer cells. Co-culture of TAFs plus MCF10.DCIS cells led to an increase in tumor size, development of multicellular structures, and formation of branch-like connections composed primarily of tumor cells [34]. The authors additionally found that DCIS cells preferentially migrate towards TAFs and form heterocellular contacts with TAFs driven by an IL-6 expression gradient at the invasive edge of DCIS tumor spheroids. Blockade of IL-6 reduced TAF-stimulated DCIS growth and proliferation and reduced the formation of TAF–cancer cell interconnections [34].

In addition to being overexpressed by TAFs, IL-6 is also a key regulator of TAF transition from normal fibroblasts. Lee et al. [35] determined that exposure to IL-6 was sufficient to induce Twist1 expression in primary human gastric fibroblasts and convert them into TAFs via phosphorylation of STAT3. Similarly, Giannoni et al. determined that prostate cancer-derived IL-6 induces the activation and increased expression of FAP by normal human prostate fibroblasts, which, in turn, leads to autocrine-activated fibroblast production of IL-6 and increased prostate cancer cell invasiveness via EMT [36]. Yeh et al. [37] additionally found that the co-culture of normal fibroblasts with bladder cancer cells increased both total IL-6 expression and bladder cancer invasiveness. Blockade of total IL-6 reduced the amount of fibroblast-mediated bladder cancer invasion, suggesting that IL-6 in the tumor microenvironment is necessary for TAF activation, which subsequently regulates cancer cell invasion [37]. Finally, IL-6 mediated signaling has also been shown to contribute to TAF-triggered therapeutic resistance. Upregulation of mammalian target of rapamycin (mTOR)–4E-BP-1 signaling has been shown to be highly activated in primary human TAFs isolated from pancreatic ductal adenocarcinoma [38]. When mTOR–4E-BP-1 signaling is inhibited, the production of alpha-SMA-positive TAFs is substantially reduced, as is the production of downstream secreted proteins, including IL-6. In murine xenografts composed of either MIA PaCa-2 or Panc-1 human pancreatic cancer cells plus CAFs, pre-treatment of TAFs with pasireotide (an inhibitor of the mTOR–4E-BP-1 pathway) followed by treatment with gemcitabine led to a reduction in tumor growth and chemoresistance [38]. Further experiments revealed that pasireotide plus gemacitabine bi-therapy was synergistic and led to gemcitabine-induced cancer cell apoptosis. Tumor collagen deposition was also reduced, suggesting that pasireotide may act as an anti-fibrotic agent [38].

#### CXCL1 (GRO-alpha)

In addition to IL-6, TAF overexpression of CXCL1 (GRO-alpha) has been recently shown to induce tumor cell invasion and enhance chemotherapeutic resistance. CXCL1 is known to promote breast tumor growth, metastasis, as well as chemotherapeutic resistance, is associated with poor patient prognosis, and is also dysregulated in melanoma, bladder cancer, and prostate cancer [39]. Increased expression of CXCL1 in TAFs has additionally been correlated with poor patient outcome and is associated with decreased expression of TGF-beta [39]. Fang et al. [39] determined that CXCL1 expression was decreased in breast cancer-derived TAFs upon treatment with TGF-beta, via Smad2/3 signaling. Regulation of Smad2/3 was also associated with decreased expression of hepatocyte growth factor (HGF), which also decreased CXCL1 expression through c-Met receptor signaling. Additionally, HGF/c-Met signaling was found to be necessary for activation of NF-kappa-B, and thus CXCL1 expression, in TAFs [39].

#### TNF-alpha and IL-1beta

The activation of TAFs from MSCs in breast cancer, specifically, has been shown to occur via high levels of TNF-alpha and IL-1beta in the tumor microenvironment. These chemokines are also known to regulate the expression of the pro-inflammatory cytokines MCP-1 (monocyte chemotactic protein-1; CCL2), IL-8 (CXCL8), and RANTES (CCL5) in breast cancer [40]. Katanov et al. [40] discovered that, when treated with TNF-alpha and IL-1beta, MSCs and TAFs derived from human breast cancer patients increased their production of MCP-1, RANTES, and IL-8. MSC treatment with TNF-alpha also resulted in increased MCP-1 expression, which subsequently induced chemoattraction of monocytic immune cell infiltrate, including tumor-associated macrophages [40]. Similarly, Taguchi et al. [41] determined that TAFs co-cultured with breast cancer cells increase their production of TNF-alpha, leading to increased production of metalloproteinase (MMP)-9 and neovascularization in the tumor microenvironment. Other groups have found that expression of IL-1beta by immune cells induces the transition of normal fibroblasts to TAFs. Once activated, TAFs then increase their production of IL-1beta, which in turn leads to the activation of the phosphoinositide 3-kinase (PI3K) and NF-kappaB pathways, both of which enhance tumor angiogenesis and growth [19].

### TASCs and microRNAs

#### microRNA dysregulation in TAFs

microRNAs (miRs) are a class of small, single-stranded, noncoding RNAs that are involved in gene expression, stem cell maintenance, cell fate determination, and developmental processes [42]. Dysregulation of miR expression has been implicated in tumor cell promotion, migration, and invasion [42]. Moreover, miR expression has been recently found to be altered in TAFs, correlating with increased tumor aggressiveness and poor patient prognosis. In a study performed by Kadera et al. [43], the UCLA 153 pancreatic ductal adenocarcinoma patient tissue microarray and 23 patient-matched lymph node metastases were analyzed via in situ hybridization for the expression of miR-21, a miR known to be involved in cancer progression. The authors discovered that miR-21 was upregulated in the TAFs of 78 % of patient tumors. Among these, high miR-21 expression was found to be correlated with decreased overall patient survival as well as patient lymph node invasion and metastasis [43].

Furthermore, activation of TASCs has been suggested to be regulated by miR-21/miR-221 in pancreatic cancer. Specifically, Ali et al. [44] analyzed the expression of miR-21/miR-221 in the conditioned medium of both human TAF cells and pancreatic stellate cells and compared it to the expression of miR-21/miR-221 in the conditioned medium of human pancreatic cell lines COLO-357 and MIAPaCa-2. Expression levels of miR-21/miR-221 were found to be higher in both the TAF and pancreatic stellate cells compared with MIAPaCa-2 pancreatic cancer cells or normal human stellate cells. Upon co-culture with TAF or pancreatic stellate cells, COLO-357 pancreatic cancer cells exhibited increased pancreatosphere formation and clonogenicity [44].

Dysregulation of miRs has also been found to impact common signaling cascades in TAFs, such as the mitogen-activated protein kinase (MAPK)/ERK pathway. Miller et al. [45] identified a novel miR signature associated with hyperactive MAPK signaling that was significantly correlated with a poor response to hormone therapy in both luminal A and B estrogen receptor (ER)-positive breast cancer subtypes. In a follow-up study, the authors determined that TAF-derived miR221/222 elicited breast cancer cell ER repression and, thus, decreased therapeutic response [46]. Furthermore, miR-15a and miR-16, which regulate cell cycle and anti-apoptotic genes, have been shown to be downregulated in the stroma of human prostate tumors [47]. In a study by Musumeci et al. [47], downregulation of miR-15a and miR-16 in the TAFs of prostate cancer cells was found to promote both prostate cancer cell growth and progression. Enhanced expression of miR-15a and -16 in TAFs decreased tumor cell proliferation and reduced the tumor-supportive capacity of the TAFs both in vitro and in vivo, suggesting that these miRs regulate the pro-tumorigenic properties of tumor-associated stroma.

Recent evidence suggests that epigenetic gene silencing due to CpG island methylation may be responsible for miR dysregulation in TAFs. A study by Li et al. [33] investigated whether DNA hypermethylation of a miR-149 was correlated with altered gene expression in gastric cancer. The authors identified a GC-rich region upstream of the miR-149 start site which was hypermethylated in TAFs compared with normal fibroblasts. Furthermore, the authors found that prostaglandin E2, an inflammatory mediator in cancer, triggered DNA methylation of the *miR-149* gene, leading to a reduction in miR-149 expression [33]. Interestingly, while hypomethylation of miRs is frequent in cancer [48], no examples of hypomethylation as a possible mechanism of miR dysregulation in TAFs were found.

#### miRs are capable of transitioning normal fibroblasts into TAFs

In addition to miR dysregulation in TAFs, miRs have also been shown to be involved in TAF transition from MSCs. Pang et al. [49] found that, upon delivery of microvesicles expressing miR-155, normal pancreatic murine fibroblasts converted into a TAF-like cell. In addition, the authors determined that microvesicles derived from pancreatic cancer cells overexpressed miR-155, indicating that pancreatic cancer cells may co-opt normal fibroblasts, transitioning them into TAFs via production of microvesicles containing miR-155 [49]. In a similar study, normal human omental fibroblasts were triple transfected with anti-miR-31, anti-miR-214, and pre-miR-155 [50]. This resulted in enhanced fibroblast migration, invasion, and colony formation, suggesting fibroblast transformation into TAFs. On the other hand, when ovarian cancer cell-associated fibroblasts were triple transfected with pre-miR-31, pre-miR-214, and anti-miR-155, the fibroblasts exhibited reduced migration, reduced invasion, as well as reduced colony formation [50]. Dysregulation of miR-210 has also been shown to convert fibroblasts into TAF-like cells. In a study by Taddei et al. [51], overexpression of miR-210 in human-derived fibroblasts was found to transition the fibroblasts into TAF-like cells able to promote prostate cancer progression via EMT as well as support prostate tumor angiogenesis.

#### miRs are involved in the recruitment and modification of stromal cells by tumor cells

In addition to being involved in the transition of fibroblasts to TAFs, miRs have been found to promote stromal cell recruitment by tumor cells in the microenvironment. In one study, the miR pair miR-126/miR-126* was found to suppress MSC recruitment into the tumor stroma of breast cancer cells [52]. This phenomenon also led to inhibition of lung metastases in a mouse xenograft model via miR-126/miR-126* inhibition of stromal-derived factor-1 alpha and CCL2 [52]. In another study, miR-149 was identified as a critical driver for the recruitment of normal fibroblasts in the tumor microenvironment in a human and mouse model of gastric cancer. Hypermethylation of the miR-149 promotor via cyclooxygenase-2/prostaglandin E2 and IL-6-mediated signaling led to repression of miR-149 expression in both human and murine fibroblasts in vitro and in vivo. Reduced expression of miR-149 was then found to promote gastric cancer progression via increased fibroblast activation and tumor cell EMT [33].

### TAFs and exosomes

Exosomes are microvesicles ranging in size from ~30–200 nm that are produced by cells and contain the molecular constituents of their cell of origin, including protein, RNA, and DNA [46]. Recent literature has focused on exosome production by TAFs and the role that these microvesicles play in signaling within the tumor microenvironment. In a study by Shah et al. [46], conditioned media from TAFs derived from three different PAM50 subtypes of breast cancer (basal, Her2+, and luminal A subtypes) were analyzed for the presence of exosomes. Transmission electron microscopy as well as nanoparticle tracking analysis confirmed the presence of exosomes produced by the TAFs as well as the uptake of these exosomes by ER-positive MCF-7/ltE2-negative breast cancer cells. TAF-derived exosomal uptake resulted in ER repression in ER-positive breast cancer cells, corresponding with increased disease recurrence and reduced overall patient survival [46]. Furthermore, Luga and Wrana [53] determined that exosomes in the conditioned media of both L-cell fibroblasts as well as TAFs derived from human breast cancer tissue stimulated the protrusive activity, motility, and lung metastasis via the Wnt-planar cell polarity pathway of MDA-MB-231 and SUM-159PT breast cancer cells, among others. Moreover, in subsequent studies, the authors showed that, when taken up by breast cancer cells, fibroblast-derived exosomes co-localize with breast cancer cell-derived Wnt11 within endocytic vesicular structures. This action was found to be important for enhancing exosome-stimulated motility in MDA-MB-231 and SUM159PT breast cancer cells [54].

#### Exosomes derived from cancer cells induce the transition of stromal cells into TAFs

Exosomes derived from solid tumor cells are known to be involved in inflammation, tumor progression, angiogenesis, and metastasis [53]. It is becoming increasingly evident that exosomes derived from tumor cells also impact the local stroma, driving production of the tumor microenvironment. This same phenomenon is evident in leukemias, where exosomes have been found to be produced by chronic lymphocytic leukemia (CLL) cells. Paggetti et al. [55] determined that exosomes produced by CLL cells were taken up by local MSCs as well as endothelial cells both ex vivo and in vivo. These CLL-derived exosomes were found to contain miRs and proteins, which resulted in an inflammatory phenotype in the endothelial cells and MSCs resembling that of TAFs. Upon uptake of the CLL-derived exosomes, both the MSCs and endothelial cells exhibited increased proliferation, migration, as well as production of inflammatory cytokines. The exosome-incorporated endothelial cells specifically showed increased tumor angiogenesis both ex vivo and in vivo, suggesting that CLL-derived exosomes promote tumor progression via transitioning of local stromal cells into TAF-like cells [55].

Exosomes produced by cancer cells under hypoxic conditions have also been shown to transition stromal cells into TAFs. In a study by Ramteke et al. [56], human prostate cancer LNCaP and PC3 cells were exposed to 1 % O_2_ or 21 % O_2_ conditions and their exosomes isolated from the derived-conditioned media. Exosomes produced under conditions of 1 % O_2_ were found to be smaller in size via nanoparticle tracking analysis as well as exhibited higher levels of CD63, CD81, and MMPs, among others, compared with exosomes produced under conditions of 21 % O_2_. When LNCaP and PC3 prostate cancer cells were co-cultured with exosomes produced under conditions of 1 % O_2_, the motility, invasiveness, and prostasphere formation of the prostate cancer cells was increased, as well as alpha-SMA expression in nearby stromal cells, resulting in formation of TAF-like cells [56]. While the authors considered 1 % O_2_ to be hypoxic and 21 % O_2_ to be normoxic conditions, it should be noted that these values are at extreme ends of the scale. Even though it is difficult to put exact figures on tissue levels, it is most widely accepted that oxygen tension in the range of 1–5 % O_2_ is generally considered “hypoxic” and oxygen tension in the range of 10–21 % O_2_ is considered “normoxic”. Oxygen tensions above 21 % are typically considered “hyperoxic” [57].

#### TAFs, exosomes, and therapeutic resistance

Data have shown that TAFs play significant roles in the therapeutic sensitivity of tumors and that therapeutic targeting of TAFs results in increased chemotherapeutic sensitivity. A recent study by Hu et al. [58] discovered that, in addition to directly increasing chemoresistance themselves, TAFs are also capable of priming cancer stem cells via TAF-derived exosomes, which further decrease drug sensitivity in colorectal cancer. Specifically, CD133+ colorectal cancer-derived stem cells were treated with TAF-derived conditioned medium (CM) derived from a human patient with Duke B colorectal adenocarcinoma. Proliferation, tumor size, clonogenicity, and chemotherapeutic resistance to conventional agents 5-fluorouracil or oxaliplatin were all found to be increased in the TAF-CM treated colorectal cancer-derived stem cells. Further investigation of the CM revealed that CD81+ exosomes were present and that these exosomes were acting on the colorectal cancer-derived stem cells via Wnt3a signaling. Blockade of exosome biogenesis with GW4869 markedly decreased colorectal cancer-derived stem cell therapeutic resistance to both 5-fluorouracil and oxaliplatin, suggesting that fibroblast-derived exosomes act on colorectal cancer-derived stem cells via the Wnt3a signaling pathway to increase drug resistance [58]. Thus, this evidence as a whole suggests that exosomes mediate reciprocal crosstalk between tumor-associated stromal cells and cancer cells during formation of the tumor microenvironment.

## Conclusions

It is becoming increasingly evident that the tumor microenvironment is a heterogeneous mixture of tumor cells plus endogenous host stroma that co-evolve during the course of disease progression. Importantly, cells of the stromal compartment of tumors, which include MSCs, fibroblasts, endothelial cells, pericytes, adipocytes, and immune cells, are increasingly being recognized as crucial players in the development of the tumor microenvironment, metastasis, immune infiltration and inflammation, and chemotherapeutic resistance (Table 1). Furthermore, one looming question that has yet to be addressed is the idea of a hierarchy, or continuum of TASCs during the transition from “normal” cell to reactive stromal cell. During this evolution, are different markers turned on/off along the way? Our work suggests that TASCs are composed of discreet subpopulations, identified by specific markers, indicating different “stages” of TASC evolution during disease progression [1] (unpublished data) (Fig. 2). And yet, within these “stages”, it is still unknown if the markers expressed during the course of transition are impacted by the cell of origin (i.e., different transition markers for fibroblasts transitioned to TAFs versus adipocytes transitioned to CAAs)? Once determined, is it possible to use these markers to identify TASCs in patient biopsies? As is evidenced by the various roles TASCs play in tumor progression, it is highly probable that any TASC population is a heterogeneous mixture of not only reactive stroma (i.e., TAFs plus CAECs), but also unactivated bystander normal cells (i.e., fibroblasts and endothelial cells). With many of the same markers being expressed by “activated” stroma as well as normal cells, what criteria will be used in the clinic to determine the margin of the tumor microenvironment during excision? Moreover, it is possible that this stromal cell population evolves with the tumor, possibly changing composition during tumor progression. How will the evolution of the stromal cell population and, conversely, additional stromal cell recruitment during the evolution of tumor progression be prevented clinically? And, how will cell-derived factors, including miRs and exosomes, be specifically targeted to prevent additional endogenous stromal cell recruitment, transition, or therapeutic resistance? Given the extensive crosstalk between tumor cells, local endogenous stroma, and tumor-associated stroma, personalized multi-modal therapeutic strategies need to be developed that target not only the tumor bulk but also the tumor-associated stromal compartment and affiliated cell-derived factors described herein.

**Table 1:** Sources of stromal cell regulation in the tumor microenvironment

	Dysregulation in TASCs	Regulator of TAF transition	Regulator of stromal cell recruitment	Mediates TASC-induced therapeutic resistance
IGFBP-2	[21]	-	-	-
MMP-11	[22]	-	-	-
IL-6	[34, 35]	[34–37]	[35]	[34, 38]
IL-8	[40]	-	-	-
IL-1beta	[40]	[22, 31]	-	-
TNF-alpha	[40, 41]	[31]	-	-
NF-kappaB	[24]	-	-	-
TGF-beta	[39]	-	-	-
CXCL1/2	[24, 39]	-	-	[24, 39]
FAP	[35]	-	-	-
MCP-1	[40]	-	-	-
Twist1	-	[13, 35]	-	-
miR-21	[43]	[44]	-	-
miR-221/222	[44]	[44]	-	[46]
MAPK/ERK	[45]	-	-	-
miR-15a/16	[47]	-	-	-
miR-210	[51]	[51]	-	-
Exosomes	[53–56]	-	-	[58]
mTOR-4E-BP-1	-	-	-	[38]
miR-126/126*	-	-	[52]	-
miR-149	-	-	[33]	-
miR-155	-	[49, 50]	-	-
miR-31	-	[50]	-	-
miR-214	-	[50]	-	-

Soluble factors produced by both the cancer cells and TAFs promote homing, migration, and invasion of tumor cells; regulate stromal cell recruitment and TAF transition; and mediate therapeutic resistance. MicroRNAs (miRs) facilitate stromal cell recruitment by cancer cells, formation of TAFs, as well as tumor growth and development. Exosomes orchestrate TAF-mediated chemotherapeutic resistance within the tumor, TAF formation, and TAF-induced cancer cell invasion and metastasis. The sources of these factors are listed. The *dash* indicates that we were unable to find evidence from the literature, within page constraints, of factor involvement in the indicated type of stromal cell regulation

miR-126* is the complement of miR-126. By all accounts in the literature, miR-126* is the standard nomenclature of the microRNA.

## Abbreviations

Alpha-SMA, alpha-smooth muscle actin; CAA, cancer-associated adipocyte; CAEC, cancer-associated endothelial cell; CASC, cancer-associated stromal cell; CAF, cancer-associated fibroblast; CCL, chemokine (C-C Motif) ligand; CLL, chronic lymphocytic leukemia; CM, conditioned medium; DCIS, ductal carcinoma in situ; EMT, epithelial-to-mesenchymal transition; EndMT, endothelial-to-mesenchymal transition; ER, estrogen receptor; FAP, fibroblast activating protein; FSP1, fibroblast specific protein-1; HGF, hepatocyte growth factor; IGFBP-2, insulin-like growth factor binding protein-2; IL, interleukin; MAPK, mitogen-activated protein kinase; MCP-1, monocyte chemotactic protein-1; miR, microRNA; MMP, matrix metalloproteinase; MSC, mesenchymal stromal cell; mTOR, mammalian target of rapamycin; NF, nuclear factor; NK, natural killer; TAF, tumor-associated fibroblast; TAM, tumor-associated macrophage; TASC, tumor-associated stromal cell; TGF, tumor growth factor; TNF, tumor necrosis factor; T_reg_, T regulatory cell; VEGF, vascular endothelial growth factor
